# Separation from the Dam Causes Negative Judgement Bias in Dairy Calves

**DOI:** 10.1371/journal.pone.0098429

**Published:** 2014-05-21

**Authors:** Rolnei R. Daros, João H. C. Costa, Marina A. G. von Keyserlingk, Maria J. Hötzel, Daniel M. Weary

**Affiliations:** 1 Animal Welfare Program, Faculty of Land and Food systems, University of British Columbia, Vancouver, Canada; 2 Laboratório de Etologia Aplicada e Bem-Estar Animal, Departamento de Zootecnia e Desenvolvimento Rural, Universidade Federal de Santa Catarina, Florianópolis, Brazil; Université Pierre et Marie Curie, France

## Abstract

Negative emotional states in humans are associated with a negative (pessimistic) response bias towards ambiguous cues in judgement tasks. Every mammalian young is eventually weaned; this period of increasing nutritional and social independence from the dam is associated with a pronounced behavioural response, especially when weaning is abrupt as commonly occurs in farm animals. The aim of the current study was to test the effect of separation from the cow on the responses of dairy calves in a judgement task. Thirteen Holstein calves were reared with their dams and trained to discriminate between red and white colours displayed on a computer monitor. These colours predicted reward or punishment outcomes using a go/no-go task. A reward was provided when calves approached the white screen and calves were punished with a timeout when they approached the red screen. Calves were then tested with non-reinforced ambiguous probes (screen colours intermediate to the two training colours). “GO” responses to these probes averaged (± SE) 72±3.6 % before separation but declined to 62±3.6 % after separation from the dam. This bias was similar to that shown by calves experiencing pain in the hours after hot-iron dehorning. These results provide the first evidence of a pessimistic judgement bias in animals following maternal separation and are indicative of low mood.

## Introduction

Young farm animals are often separated from the dam far earlier than what occurs under natural conditions. For example, dairy calves are typically separated from the cow within hours of birth. When calves are allowed to stay with the cow a strong bond develops [1], and this bond persists even after short periods of separation [2]. The cow-calf bond typically weakens as the calf becomes less reliant on milk [3], [4]. Early separation interferes with the development of the cow-calf bond and thus helps to reduce the distress response when separated [5], [6]. On some farms calves may be separated days, weeks or months after birth; in these cases abrupt weaning results in an intense behavioural and physiological response [7]–[9].

Weaning from the dam typically involves both the loss of access to milk and the breaking of the social bond, although in some experiments and weaning protocols these elements have been separated (see reviews [1], [10]). The distress responses by cow and calf after weaning are typically mitigated if separation occurs days after the calf is no longer nutritionally dependent upon the cow’s milk. For example, Haley et al. [7] applied nose flaps to calves that prevented suckling but allowed full social contact with the cow; these calves vocalized much less when later separated from the cow than did control calves that were allowed to suckle right up to the time of separation.

The behavioural and physiological responses described above are indicative of an acute emotional response to separation from the dam. Modern definitions of emotion include behavioural, physiological, cognitive, and subjective components [11]. Direct assessment of the subjective component is difficult in animals, as they are unable to verbally convey their subjective experiences. Recent studies have begun to address the cognitive component. Studies on humans have shown that several aspects of cognitive functioning, including attention and memory, are affected by emotional experiences [11]. One special area of attention has been judgement tasks; for example, depressed patients typically interpret ambiguous stimuli more negatively than do people in positive emotional states [12].

Harding et al. [13] were the first to use this paradigm to test judgement bias in animals. Rats were trained to press a leaver in response to a tone associated with a positive event and to not press the leaver in response to a second tone associated with a negative event. Once trained, three ambiguous tones intermediate in frequency to the two training tones were introduced to determine if rats responded to these ambiguous stimuli as positive or negative. Judgement bias tasks have been applied to a range of non-human animals (e.g. starlings [14], sheep [15], rats [16], honeybees, [17], pigs [18], monkeys [19]), including dairy calves [20]. Negative judgement biases (i.e. responding negatively to ambiguous stimuli) have been reported for animals experiencing negative states such as those associated with exposure to unpredictable environmental changes [13] and chronic stress [21].

No research to date has assessed the effect of maternal separation on any aspect of cognition. The primary aim of the current study was to compare calf responses to ambiguous stimuli before and after separation from the dam at 42 days of age. We predicted that the negative emotional state associated with separation would result in a negative judgement bias towards ambiguous cues. Another recent study has shown that calves show a negative judgement bias following the routine procedure of hot-iron dehorning [20]. Thus a secondary aim was to also assess the judgement bias is response to hot-iron dehorning, and thus compare the known bias due to pain with that due to separation from the dam.

## Methods

### (a) Animals and Housing

We used 13 male Holstein calves and their dams housed at the University of British Columbia Dairy Education and Research Centre (Agassiz, BC, Canada). The experiment was carried during the autumn of 2012. This study was approved by the Canadian Council on Animal Care (Protocol number: A12-0337).

Calves were kept with their dams in a calving pen for approximately 24 h after birth; cow and calf were then moved together to a dynamic group, varying in size from 4 to 8 cow-calf pairs over the course of the study. The experimental area included a pen with 12 sand-bedded free stalls and a sawdust bedded creep area. Calves had free access to cows in the entire area from 19.00 h to 07.00 h but were otherwise restricted to the creep. Calves were able to physically interact with the cow across the barrier separating the creep area from the free stall pen. Throughout the experiment cows were fitted with udder nets that prevented calves from suckling. Pasteurized whole milk was provided to calves in a bottle twice a day, 8 L/d for the first 28 d of age, and 6 L/d until weaning from milk at 56 d. Calves had free access to a calf starter, to a total mixed ration and to water throughout the study. Calves underwent health checks weekly and calves identified as ill were treated according to the farm’s standard veterinary procedure.

### (b) Training

**Figure 1:**
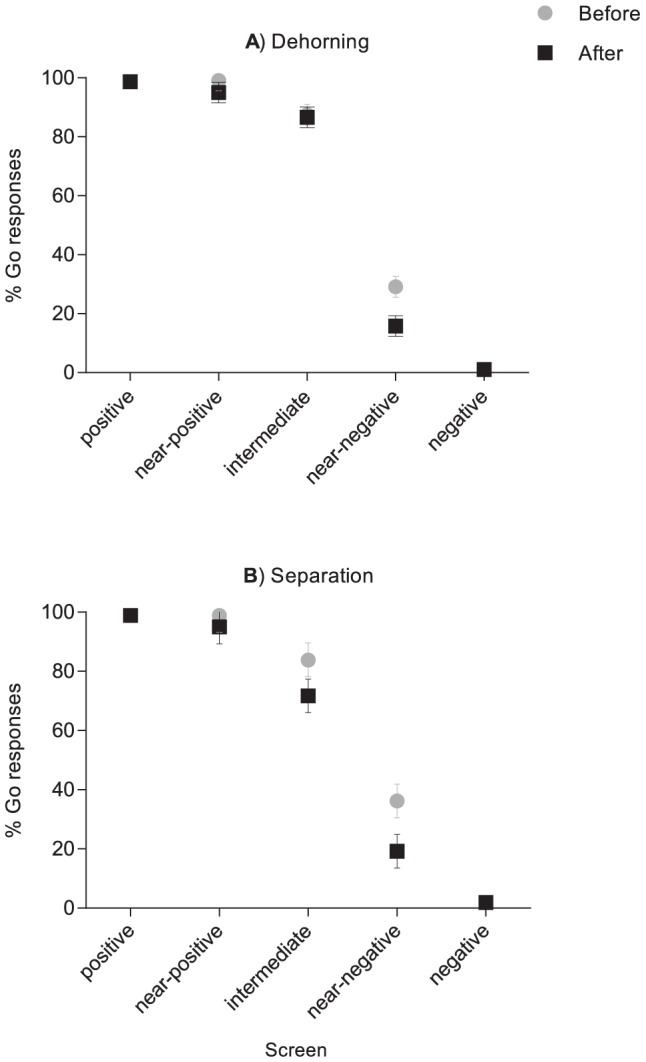
Mean±SE % GO responses to screens presented during test sessions before and after a) dehorning (n = 12 calves) and b) separation from the dam (n = 8 calves). Responses are shown separately for the two training screens (positive and negative) and for the three ambiguous probe screens (near-positive, intermediate and near-negative).

Calves received their entire daily allotment of milk during daily training and testing sessions. Twice daily training sessions were conducted in a training pen (1.2×2 m; for a diagram see Figure 1 in our previous work Neave et al. [20]) at 07.00 and 16.30 h, starting at 5 d of age. Calves were trained using a go/no-go task to discriminate two colours (red and white) displayed on a 15” LCD monitor. Our previous work alternated between red and white as the positive cue and found no effect (see Neave et al. [20]). To simplify the methodology in the present experiment we elected to use the white as the positive cue for all calves. Also, given that we tested the change in responses to probes within subject (e.g. before vs. after separation), there was no advantage in varying the positive training cue. “Go” responses (approaching to within 20 cm of the screen) to the white screen (positive cue) were rewarded with milk. To receive the reward calves were required to turn around and approach the milk feeder at which time the researcher placed the nipple bottle in the holder so that the calf was able to access the teat. Go responses to the red screen (negative cue) was punished with a 1-min delay to the next trial signalled by a whistle (97 dB, C weighting, as measured at 1 m using a Realistic sound-level meter, model 33–2050). Initial training sessions consisted of 20 trials with positive cues only. After reaching a criterion of 90% correct responses (i.e. approaching the screen following the cue) in 3 consecutive sessions, the calves were gradually introduced to the negative cue (increasing from 2 to 6 trials over 3 sessions). Training continued until calves reached the learning criterion of 90% correct responses for positive and 100% of correct responses for negative cues over 2 consecutive sessions. During each trial the positive and the negative cues were shown for 4 s. The number of negative cues was then increased from 6 to 8, 12, 16 and 20, these cues were randomly interspersed among the 20 positive screens, resulting in each training sessions having a total of 40 trials. Throughout the process where we increased the number of negative cues the same 90% for positive and 100% for negative learning criterion was applied. Once calves reached the learning criterion, the positive reinforcement rate was reduced from 100% to 50% over the course of 3 sessions; the negative reinforcement rate was not reduced. This practice was introduced to prevent calves from learning the ambiguous probes (described below). When calves achieved over 85% correct responses for positive and 85% correct responses for negative cues over 3 consecutive sessions they were considered fully trained and able to proceed to testing.

### (c) Testing

During each test session calves were presented with 60 screens: 23 positive, 22 negative and 15 ambiguous probes. Five probes were similar in colour to the positive training colour (i.e. 25% red), 5 were intermediate between the positive and negative training screens (i.e. 50% red) and 5 were similar to the negative screens (i.e. 75% red). Colours were generated using Adobe Photoshop Elements [22] by adjusting the saturation level of 100% red. The sequence of screens shown was pseudo randomized: probes were never displayed consecutively, the same training cues were never presented more than 2 times in sequence, a positive trial (scheduled to be rewarded contingent on a correct response) was presented at least once every four trials. A reward was only provided following a correct response to the positive cue. During testing incorrect responses to negative cues were not punished to avoid delaying test sessions and influencing calf judgement towards ambiguous probes; responses to ambiguous probe screens were neither rewarded nor punished. Test sessions were performed before and after dehorning and separation as detailed below.

### (d) Dehorning

Calves were dehorned at approximately 10.30 h when 36 d of age. Following standard farm practice calves were first sedated with an intramuscular injection of xylazine (Rompun, 2%, Bayer Inc., Ontario; 0.25 mg/kg body weight). A local anaesthetic (5 mL per side of 2% Lidocaine; Ayerst Veterinary Labs, Ontario) was then injected subcutaneously into the corneal nerve of each horn bud, located under and along the occipital groove. Five min later an electric hot-iron was applied to each horn bud for a total time of 30 s. The baseline test sessions were performed during the evening (16:30 h) and morning (07:00 h) immediately before dehorning. The post dehorning test sessions took place in the evening (16:30 h, 6 h after the procedure) and the next morning (07:00, 22 h after the procedure).

### (e) Separation

Cows were removed from the experimental pen when their calf reached 42 d of age. Following the standard routine, the cow was taken to the milking parlour at 17:30 h but was then moved to another pen (in a separate barn) after milking. Test sessions occurred the morning and evening immediately before separation (baseline) and the next 3 mornings, i.e. 12, 36 and 60 h, respectively, after separation.

### (f) Statistical Analysis

All analyses were with mixed model (Proc Mixed in SAS software [23]) specifying calf as a random effect and session*probe as a repeated measure with compound-symmetry as the co-variance structure. The original dataset is available in a public repository [24]. The dependent variable was the percentage of go responses per stimuli, per session, per calf. In preliminary analyses we compared the multiple test sessions before and after both dehorning and separation (e.g. the morning versus the evening session immediately before dehorning and the morning versus the evening session immediately after dehorning). Responses to ambiguous stimuli did not differ between test sessions that pertained to the same phase (e.g. multiple session before dehorning) so these were pooled to allow a single test of the effect of dehorning (before versus after) and separation (before versus after). We tested the effect of probe location (near the positive, intermediate or near the negative), phase (before or after), and the interaction between probe and phase. This analysis was repeated for the two training screens (positive and negative) to test if responses to the training screens differed with phase. If calves learned that responses to probes were not rewarded we would predict a decline in response rates to the probes over multiple test sessions. We tested this prediction by comparing responses to the probes before dehorning with those before separation. To test if the bias differed for dehorning and separation we also compared the sessions after dehorning with the sessions after separation.

All analyses only included calves that were assessed as clinically healthy in each of the test sessions; calves were subjected to a health exam by the researchers under the supervision of a veterinarian every week and removed from the study if they showed any signs of sickness. For the test of dehorning, 12 out of the 13 calves were included. For separation, 8 of the 13 calves were included.

## Results

All calves were able to discriminate between the positive and negative training screens, requiring, on average ± SD, 32±5.2 training sessions to achieve criterion for testing. Responses to the training cues during test sessions did not differ across periods. Response rates during baseline testing before dehorning calves averaged±SE 98±0.5% for the positive screen and 1±0.5% for negative screen (Figure 1 a); before separation the response rate averaged 99±0.5 and 2±0.5% for positive and negative screens (Figure 1 b), respectively.

Overall calves were less likely to approach ambiguous screens after dehorning (F_1,11_ = 6.4, p = 0.03), approaching 66% of ambiguous screens versus 73% before dehorning. Probe location also affected calves’ response: calves were much more likely to approach the near-positive versus near-negative probe (F_2,22_ = 371.6, p<0.001). There was no interaction between phase and probe (F_2,22_ = 2.4, p = 0.12), but numerically the bias was strongest for the near-negative probe (Figure 1 a).

Separation from the dam also affected response to probes (F_1,7_ = 6.3, p = 0.04); calves approached 72% of ambiguous screens before separation versus 62% after separation from the dam (Figure 1 b). Probe location again affected the response (F_2,14_ = 88.9, p<0.001). The interaction between probe location and phase relative to separation was not significant (F_2,14_ = 0.8, p = 0.47), but the bias was most pronounced for the intermediate and near-negative probes.

**Figure 2:**
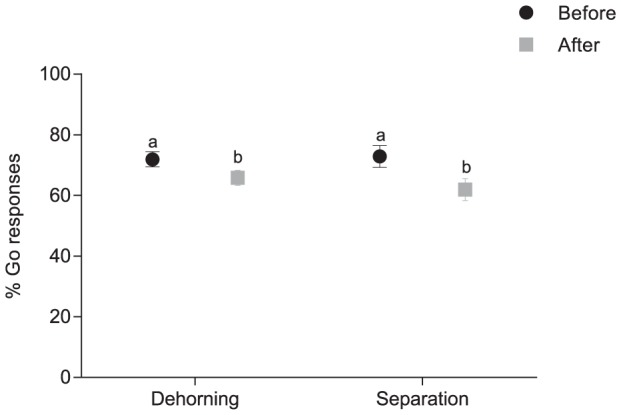
Mean±SE % GO responses to the three ambiguous probe screens (near-positive, intermediate and near-negative) in multiple sessions before dehorning and before separation from dam and multiple sessions after dehorning and after separation from dam. Different letter indicate a statistically significant difference (p>0.05).

Calf responses to probes were similar at sessions before dehorning and before separation (F_1,6_ = 0.0, p = 0.97; Figure 2). Response rates were also similar after dehorning and after separation (F_1,6_ = 1.5, p = 0.27; Figure 2).

## Discussion

Dairy calves showed a negative response bias to ambiguous cues after separation from the dam; this pessimistic response bias is consistent with a negative emotional state. This study provides the first evidence of a cognitive response to separation from the dam in any species. Previous studies in cattle have described distress-related behaviours and physiological changes following separation at different ages, such as increases in vocalization and activity (e.g. [7], [25]), and increases in cortisol and noradrenaline (e.g. [26], [27]). Our study indicates that calves show a negative judgment bias for at least 2.5 days after separation; physiological and other behavioural changes may have persisted beyond this time. Previous work has shown that the response to separation is dependent in part on the age of the calf, breed and the type of rearing system used (see reviews [5], [10]).

Weaning typically involves the loss of social contact with the mother and access to the mother’s milk, as well as changes in the social and physical environment; disentangling these factors can be a challenge [10]. Our design allowed us to terminate social contact with the dam while keeping all other factors constant including social group, physical environment, and milk supply from artificial teats. This design allows us to attribute the judgement bias response to separation from the dam.

Our study is the first to assess the effects of two different manipulations of affect on judgement bias. Previous work has shown a negative response judgment at 6 and 22 h after dehorning [20], which also coincides with other behavioural and physiological responses indicative of post-operative pain [28]. The calves in the current study also showed a negative bias after dehorning, replicating the results of Neave et al. [20] and supporting the idea that pain induces a negative judgement bias. Moreover, the magnitude of bias was similar following dehorning and separation. It is not clear if the magnitude of bias can be used as a measure of the strength of the emotional experience, but if so these results suggest that the emotional response to the two procedures is similar. In humans, physical pain and ‘social’ pain results in heightened activity in the same regions of the brain, such as the anterior cingulated cortex, using the same neural pathways [29], [30].

One explanation for reduced responding after the procedure is that calves simply learned to stop responding to the non-reinforced ambiguous cues. For example, Doyle et al. [31] reported that sheep responded less to the ambiguous cues as sessions progressed, likely as a result of learning. To prevent calves from learning we used a 50% of reinforcement rate to the positive stimuli. From our results, three lines of evidence suggest that calves did not learn to avoid the non-reinforced ambiguous cues. The first is that we found no evidence of changes in responding to these probes across multiple testing sessions before and after each procedure. We also found no evidence of a decline in responding to the test screens in the session before dehorning versus sessions before separation, and no decline after dehorning versus after separation. We conclude that the bias was due to the procedures and not to learning.

Another potential explanation for reduced responding to the probes is that calves were simply less motivated to drink milk. However, our results show that there was no difference in responses to positive (or negative) training screens after dehorning or separation, indicating that the calves’ motivation to drink milk was not affected.

Studies on human subjects have shown that anxiety-like states tend to result in judgement biases that are more pronounced at near-negative cues. In contrast, subjects experiencing depression-like states are more likely to show a near-positive probe bias [32]. Burman et al. [33] and Pomerantz et al. [19] speculated that the negative judgment bias towards near-negative cues in their animal studies meant that the negative affect was more like anxiety than depression. In the current experiment calves showed numerically stronger judgement biases towards the near-negative probe following separation and dehorning; the latter result is consistent with Neave et al. [20]. We suggest that these procedures induce a high intensity negative affect more similar to anxiety than depression.

In conclusion, separation from the dam induced a pessimistic response bias in a judgement task; this response bias was similar to that induced by dehorning. This cognitive bias suggests that calves experience an emotional response to both pain and social loss.
